# The non-pathogenic mycobacteria *M. smegmatis *and *M. fortuitum *induce rapid host cell apoptosis via a caspase-3 and TNF dependent pathway

**DOI:** 10.1186/1471-2180-10-237

**Published:** 2010-09-10

**Authors:** Amro Bohsali, Hana Abdalla, Kamalakannan Velmurugan, Volker Briken

**Affiliations:** 1 Department of Cell Biology and Molecular Genetics, University of Maryland, Campus Drive, College Park, MD, 20742, USA; 2 Maryland Pathogen Research Institute, University of Maryland, Campus Drive, College Park, MD, 20742, USA; 3 AERAS Global TB Vaccine Foundation, 1405 Research Blvd., Rockville, MD, 20850, USA

## Abstract

**Background:**

The HIV pandemic raised the potential for facultative-pathogenic mycobacterial species like, *Mycobacterium kansasii*, to cause disseminating disease in humans with immune deficiencies. In contrast, non-pathogenic mycobacterial species, like *M. smegmatis*, are not known to cause disseminating disease even in immunocompromised individuals. We hypothesized that this difference in phenotype could be explained by the strong induction of an innate immune response by the non-pathogenic mycobacterial species.

**Results:**

A comparison of two rapid-growing, non-pathogenic species (*M. smegmatis *and *M. fortuitum*) with two facultative-pathogenic species (*M. kansasii *and *M. bovis *BCG) demonstrated that only the non-pathogenic bacteria induced strong apoptosis in human THP-1 cells and murine bone marrow-derived macrophages (BMDM) and dendritic cells (BMDD). The phospho-*myo*-inositol modification of lipoarabinomannan (PI-LAM) isolated from non-pathogenic species may be one of the cell wall components responsible for the pro-inflammatory activity of the whole bacteria. Indeed, PI-LAM induces high levels of apoptosis and IL-12 expression compared to the mannosyl modification of LAM isolated from facultative-pathogenic mycobacteria. The apoptosis induced by non-pathogenic *M. smegmatis *was dependent upon caspase-3 activation and TNF secretion. Consistently, BALB/c BMDM responded by secreting large amounts of TNF upon infection with non-pathogenic but not facultative-pathogenic mycobacteria. Interestingly, C57Bl/6 BMDM do not undergo apoptosis upon infection with non-pathogenic mycobacteria despite the fact that they still induce an increase in TNF secretion. This suggests that the host cell signaling pathways are different between these two mouse genotypes and that TNF is necessary but not sufficient to induce host cell apoptosis.

**Conclusion:**

These results demonstrate a much stronger induction of the innate immune response by non-pathogenic versus facultative-pathogenic mycobacteria as measured by host cell apoptosis, IL-12 and TNF cytokine induction. These observations lend support to the hypothesis that the strong induction of the innate immune response is a major reason for the lack of pathogenicity in fast-growing mycobacteria.

## Background

Facultative-pathogenic mycobacterial species cause disseminating mycobacterial infections in humans that are defective in the acquired immune response (IR). For example, *M. kansasii *and *M. avium *are often found as opportunistic pathogens in immunosuppressed individuals due to AIDS. In contrast, non-pathogenic mycobacteria of the *M. fortuitum *and *M. smegmatis *group do not cause disseminating disease even in immunosupressed individuals[1]. Therefore, we hypothesized that the inability of non-pathogenic species to cause disease could be due to their strong capacity to induce an innate IR, which is sufficient to defend against these species of mycobacteria even in individuals with defective acquired immunity.

The capacity of infected macrophages to undergo apoptosis after infection is an efficient mechanism of innate IR against mycobacteria[2]. Indeed, the induction of apoptosis of infected macrophages may induce direct killing of intracellular mycobacteria [3,4]. In addition, mycobacteria contained in apoptotic bodies can be taken up via phagocytosis by uninfected bystander macrophages which are then able to kill the bacteria more efficiently [5]. Furthermore the importance of macrophage apoptosis for the IR was underscored by the recent findings that host susceptibility or resistance to mycobacterial infections could be linked to the capacity of the infected macrophages to undergo necrosis or apoptosis, respectively[6]. Consistently, virulent *M. tuberculosis *strains express proteins implicated in inhibiting host cell apoptosis such as the superoxide dismutase A (SodA), catalase G (KatG) and NuoG which is part of the NDH-1 protein complex. The deletion of any of these genes strongly attenuates the virulence of the bacteria suggesting that host cell apoptosis inhibition is a virulence pathway [7-9].

In primary human alveolar macrophages the facultative-pathogenic mycobacteria (*M. kansasii *and *M. bovis *BCG) induced significantly more apoptosis then four different virulent strains of *M. tuberculosis *after 5 days of infection [10]. Interestingly, *M. smegmatis *induces significant apoptosis in differentiated human THP-1 cells after only 24 h [8], suggesting the presence of potent mycobacterial ligands capable of inducing host cell signaling. The phospho-*myo*-inositol-lipoarabinomannan (PI-LAM) isolated from the cell wall of an unidentified fast-growing mycobacterial species, also referred to Ara-LAM, could be one such ligand, since it has been shown to induce host cell apoptosis [11,12].

The host cell cytokine response during mycobacterial infections is regulated by mitogen activated protein kinase (MAPK) pathways[13]. The facultative-pathogenic *M. avium *induced a profoundly different host cell signaling response when compared to the non-pathogenic *M. smegmatis *[14]. In particular, the infection with *M. smegmatis *led to an increased p38 and ERK1/2 MAPKs activity in BMDMs which was necessary for increased TNF secretion [14]. Furthermore, this increase in MAPKs was dependent upon prolonged stimulation of calmodulin/calmodulin kinase and cAMP/protein kinase A pathways [15]. In addition, sphingosine kinase, phosphoinositide-specific phospholipase C and conventional protein kinase C were all implicated in *M. smegmatis*-induced activation of Erk1/2 [16]. One downstream target of the MAPK p38 was determined to be the transcription factor cyclic AMP response element binding protein (CREB) which was more activated in *M. smegmatis*-infected cells [17].

In order to understand why non-pathogenic mycobacteria are strongly attenuated we compared their capacity to induce an innate IR to that of facultative-pathogenic mycobacteria. The induction of apoptosis and the stimulation of TNF expression in macrophages were analyzed and in both cases the macrophage response was much stronger for the non-pathogenic mycobacteria than the facultative-pathogenic mycobacteria. The induction of TNF secretion was important for the increase in caspase-3-dependent host cell apoptosis in BMDM. Furthermore, purified PI-LAM of the nonpathogenic mycobacterial species interacted with the TLR-2 and induced apoptosis and IL-12 p40 expression, whereas the purified Man-LAM of the facultative-pathogenic mycobacteria had no such activity. Altogether, facultative-pathogenic mycobacteria induce less of an innate immune response in macrophages relative to non-pathogenic mycobacteria.

## Results and Discussion

### Non-pathogenic mycobacteria induce increased host cell apoptosis

**Figure 1:**
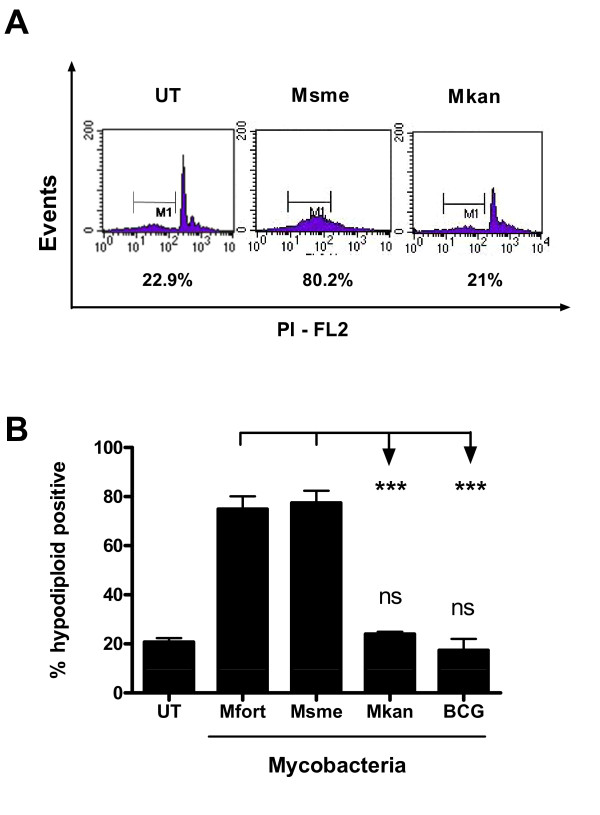
**Differences in apoptosis induced by facultative-pathogenic versus non-pathogenic mycobacteria in primary murine macrophages**. Differentiated BALB/c BMDMs were infected at an MOI of 10:1 with *M. smegmatis *(Msme), *M. fortuitum *(Mfort), *M. kansasii *(Mkan), *M. bovis *BCG or left untreated (UT). The percentage of apoptotic cells was determined using a propidium iodide based staining protocol to detect the population of hypodiploid cells via flow cytometry at 20 h after infection. Representative histograms are shown in **A. B**. The average and standard deviation of three independent experiments is shown. For this and all subsequent figures asterisks indicate statistically significance with * = 0.05>p > 0.01, ** = 0.01>p > 0.001 and *** = p < 0.001 which was determined by using one way ANOVA using GraphPad Prism5.0 software.

In order to test the apoptotic response of macrophages following infection with facultative-pathogenic compared to non-pathogenic mycobacteria, we used bone marrow-derived macrophages (BMDM) from BALB/c mice and infected them with *M. smegmatis, M. fortuitum*, *M. bovis *BCG, or *M. kansasii *for two hours. We then incubated the macrophages in infection medium with gentamycin for an additional twenty hours. The percentage of apoptotic cells was determined by quantifying the fraction of hypodiploid positive cells via flow cytometry (Figure 1A). 75-80% of BMDMs infected with *M. smegmatis *and *M. fortuitum *were hypodiploid positive which was significantly different (p < 0.001) from BMDMs infect with facultative-pathogenic mycobacteria (Figure 1B). Indeed, BMDMs infected with BCG and *M. kansasii *did not show any significantly increased levels of apoptosis compared to the untreated control cells during the course of this short term infection (p > 0.05; Figure 1B).

**Figure 2:**
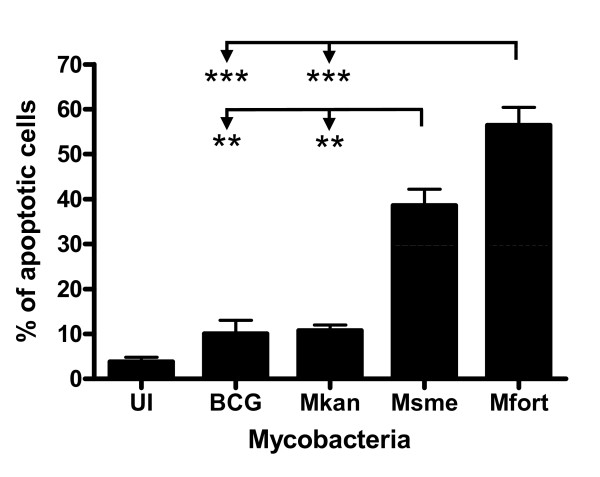
**Difference in apoptosis induction between facultative and non-pathogenic mycobacteria in a human macrophage cell line**. PMA-differentiated THP-1 cells were infected with indicated mycobacteria and the amount of apoptosis was determined 20 h after infection using TUNEL assay and flow cytometry on duplicate samples. The results are the mean and standard deviation of three independent experiments.

This difference in host cell apoptosis induction is conserved in human macrophage-like cells (THP-1 cell line) which are a good model for the behavior of primary human alveolar macrophages in response to mycobacterial infections[18]. PMA-differentiated THP-1 cells were infected and incubated for an additional 20 h at which time the percentage of apoptotic cells was determined using the TUNEL assay as previously described[8]. Figure 2 shows that *M. smegmatis*-infected cells underwent about a 4 fold increase in apoptosis (~40% total, p < 0.005) and *M. fortuitum *infection resulted in a 5-6 fold increase (~55% total, p < 0.001) when compared to cells infected with facultative pathogenic mycobacteria (~10%) (Figure 2). This difference in apoptotic response between non-pathogenic and facultative-pathogenic mycobacteria supports our hypothesis that non-pathogenic mycobacteria induce a very potent innate immune response when compared to facultative-pathogenic mycobacteria.

The induction of macrophage apoptosis has been implicated in innate host defense against mycobacteria[2]. The importance of apoptosis in innate immune response was demonstrated by the attenuation of a pro-apoptotic Mtb mutant in immunodeficient SCID mice [8]. In a previous study it was demonstrated that facultative-pathogenic mycobacteria (*M. kansasii *and *M. bovis *BCG) induce more apoptosis then virulent mycobacteria in primary alveolar macrophages after five to seven days of infection[10]. Interestingly, we demonstrated that *M. smegmatis *induces apoptosis of THP-1 cell already after 16 h of infection[8]. The current results thus extend this initial observation to another fast-growing, non-pathogenic mycobacterial species. They indicate that the pro-apoptotic capacity might be a general characteristic of this group of mycobacteria but it would clearly be desirable to analyze more strains of mycobacteria in order to support this generalization.

### The PI-LAM cell wall component of non-pathogenic mycobacteria mediates pro-inflammatory response

**Figure 3:**
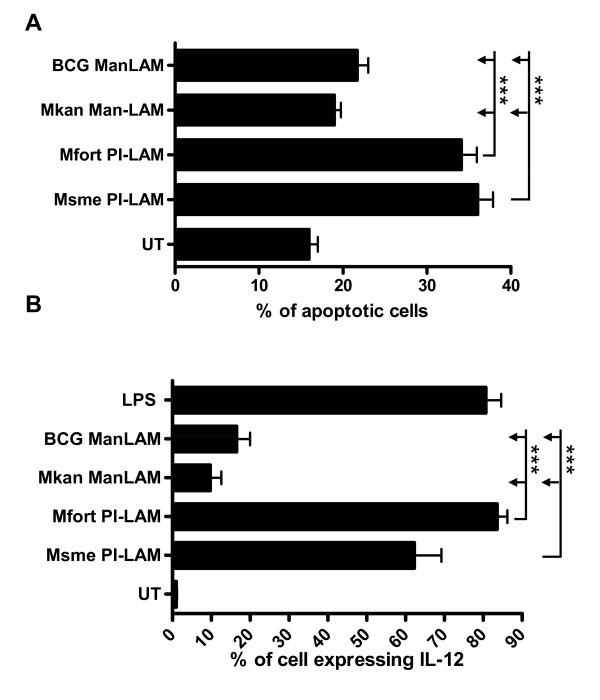
**PI-LAM of fast-growing mycobacteria induces apoptosis and IL-12 gene expression in macrophages**. **A**. Differentiated human THP-1 cells were not treated (UT) or incubated with the indicated lipoglycans at 20 μg/ml for 24 h. The percentage of apoptotic cells was determined as Annexin-V-Alexa488-positive and propidium iodide-negative cells out of 10,000 analyzed cells by flow cytometry. **B**. The induction of Il-12 gene expression was analyzed by incubating a murine macrophage (RAW/pIL-12-GFP) reporter cell line which has the IL-12p40 promoter in front of the GFP gene, with the indicated lipoglycans for 16 h. GFP-expression was analyzed on 5,000 cells and the mean and standard deviation of three independent experiments is shown.

Pathogen associated molecular patterns (PAMP) interact with pathogen pattern recognition receptors (PRR) to induce host immune responses[19]. Toll-like receptors bind to bacterial and viral derived ligands and may induce host cell apoptosis [20,21]. The mycobacterial cell wall contains several components with immunomodulatory activities [22,23]. In particular, lipoarabinomannan (LAM) and its differential terminal modifications with mannose caps (Man-LAM) versus phosphomyo-inositol caps (PI-LAM) have been extensively investigated [24,25]. Nevertheless, the PI-LAM (named Ara-LAM) in most previous studies used was derived from an unidentified, fast-growing mycobacterium[26]. Here we extended the analysis to include two PI-LAMs, kindly provided by Drs. J. Nigou and G. Puzo, purified from the non-pathogenic, fast-growing *M. smegmatis *and *M. fortuitum *[27]. THP-1 cells were treated with 20 μg/ml of the different LAMs for 24 h and the percentage of apoptotic cells was determined using Annexin-V assay as previously described [12]. The PI-LAM of both non-pathogenic mycobacteria induced approximately a twofold increase in apoptosis (~35-40%) when compared to the Man-LAM from the facultative-pathogenic mycobacteria (~20%) which was a significant difference with p < 0.001 (Figure 3A). In addition, the pro-inflammatory potential of the PI-LAMs was analyzed using an IL-12 p40 reporter cell line[12]. The p40 promoter was activated in 60-80% of the cells treated with PI-LAM when compared to only 10-20% of the cells treated with either Man-LAM (p < 0.001; Figure 3B). The induction of the IL-12 reporter by the PI-LAMs was similar to the promoter activity induced by LPS (~80%), a well-characterized TLR-4 ligand that efficiently induces IL-12 secretion.

**Figure 4:**
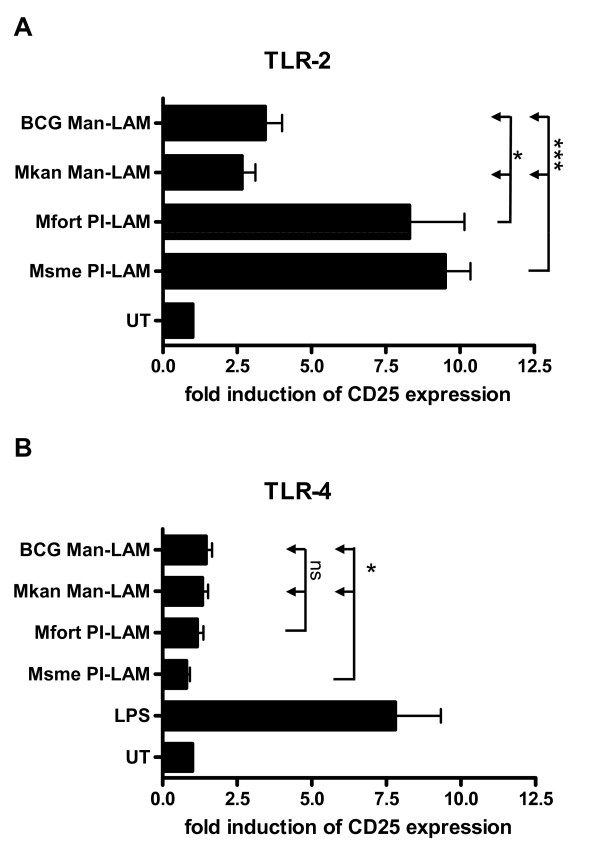
**PI-LAMs activate cells in a TLR-2-dependent manner**. **A**. CHO/CD14/TLR-2 and **B**. CHO/CD14/TLR-4 reporter cell lines were incubated with the indicated lipoglycans at 20 μg/ml or LPS at 1 μg/ml for 16 h. Cellular activation was measured by determining the expression of CD25 at the cell surface by using anti-CD25 monoclonal antibodies and flow cytometry. The mean fluorescence intensities were determined and the fold induction over untreated cells was calculated and the mean and standard deviation of three independent experiments is shown.

Another reporter cell line was used to study the interaction of PI- and Man-LAM with TLR-2 and TLR-4 [28]. In CHO cells, transfected with either human TLR-2 or TLR-4, the induction of TLR signaling was measured by flow cytometry via cell surface staining of the CD25 molecule which is under control of a promoter inducible by TLR-2 and TLR-4 signaling (Figure 4) [28]. The PI-LAMs both induced an 8 to 10 fold increase in CD25 expression when compared to untreated cells, whereas both Man-LAM species only induced maximal 3 fold inductions (p < 0.05 for Msme PI-LAM and p < 0.001 for Mfort PI-LAM; Figure 4A). All of the LAMs had minimal interaction with TLR-4 (less than 2 fold induction), when compared to LPS-treated cells which increased CD25 expression about 7 fold (Figure 4B).

Overall, the results of the current study are very consistent with reported results demonstrating that the PI-LAM of an unidentified, fast-growing mycobacterial species induces host cell cytokine secretion and apoptosis [24]. We extended these results to include PI-LAM of *M. smegmatis *and another PI-LAM of *M. fortuitum *[27], both of which induced host cell apoptosis and cytokine secretion. These results thus confirmed the general principle that PI-modified LAMs are pro-inflammatory. Furthermore, both of these PI-LAMs interact with macrophage TLR-2 but not TLR-4 receptors suggesting that the PI-component is the ligand of the TLR-2. Interestingly, despite the existence of a mycolic acid rich outermembrane in myocbacteria, it seems that LAM are still able to reach the outermost layers of the envelop to be exposed at the cell surface of the bacterium and thus exert their function as immunomodulins [29-31].

### Non-pathogenic mycobacteria induce apoptosis via TNF and caspase-3 signaling pathways

TNF is a central pro-inflammatory cytokine that mediates and regulates innate immunity. TNF binding to TNF-R1 may lead to activation of NF- B, followed by gene transcription, production of inflammatory mediators and survival proteins. On the other hand, TNF binding may also initiate JNK protein kinase activation followed by activation of caspase-8 and downstream effector caspases such as caspase-3 resulting in apoptosis of the cell [32].

**Figure 5:**
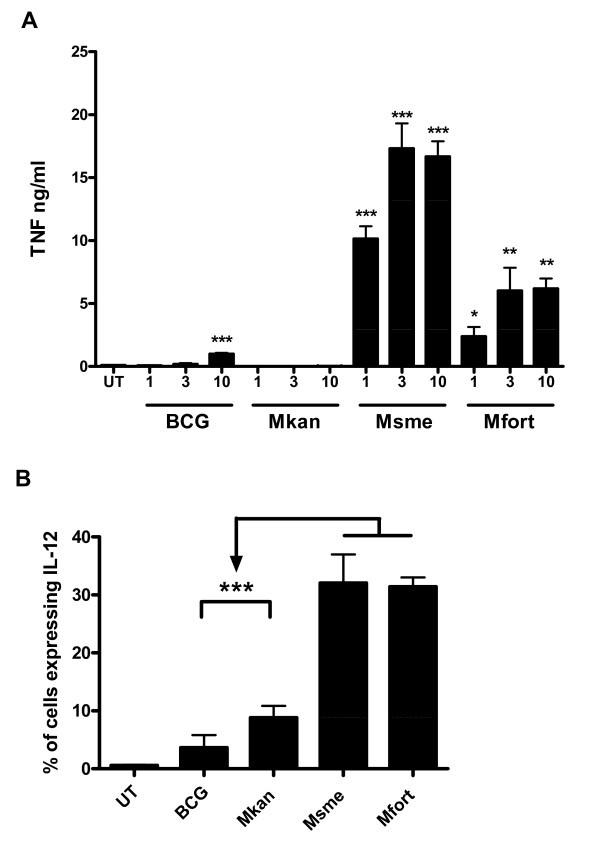
**Differences in TNF secretion and IL-12 induction between facultative-pathogenic and non-pathogenic mycobacteria infected macrophages**. **A**. BALB/c BMDMs were infected at MOIs of 1:1, 3:1, and 10:1 with *M. smegmatis *(Msme), *M. fortuitum *(Mfort), *M. kansasii *(Mkan), *M. bovis *BCG, or left untreated (UT). Cells were infected in triplicates for 2 h then washed and incubated in infection media with 100 μg/ml gentamycin for an additional 20 h. Culture supernatants were then collected and the amounts of secreted TNF was determined using ELISA. The values are the mean and standard deviation of triplicate readings and they are representative of three independent experiments. **B**. The induction of Il-12 gene expression was analyzed by infecting RAW/pIL-12-GFP macrophages with the indicated bacteria for 2 h at an MOI of 10:1. The GFP-expression was analyzed on 5,000 cells 16 h later and the mean and standard deviation of three independent experiments is shown.

In order to analyze the importance of TNF in apoptosis induction by the non-pathogenic mycoabcteria BALB/c BMDMs were infected with *M. smegmatis*, *M. fortuitum*, BCG, and *M. kansasii *at three MOIs (1:1, 3:1, and 10:1) for two hours and then incubated in medium with gentamycin for an additional 20 hours. The amounts of secreted TNF in the culture supernatants were measured using ELISA. BALB/c macrophages infected with *M. smegmatis *secreted 10 to 18 fold more TNF than macrophages infected with BCG or *M. kansasii*, which did not secrete significant amounts of TNF. *M. fortuitum *infected macrophages also secreted around 6 fold more TNF than BCG and *M. kansasii *infected cells (Figure 5A). The impact of non-pathogenic mycoabcteria on IL-12 gene expression was also much higher when compared to facultative-pathogenic mycobacteria (Figure 5B). Indeed, infection of the IL-12 p40 reporter cell line [12] at an MOI of 10:1 with *M. smegmatis *or *M. fortuitum *resulted in p40 promoter-driven GFP expression in about 30% of the cells, whereas only 5-10% of the cells became GFP positive after infection with the facultative-pathogenic mycobacteria (p < 0.001, Figure 5B). In conclusion, our results demonstrate a stronger induction of two pro-inflammatory cytokines (TNF and IL-12) after macrophage infection with two species of non-pathogenic mycobacteria when compared to facultative-pathogenic mycobacteria.

**Figure 6:**
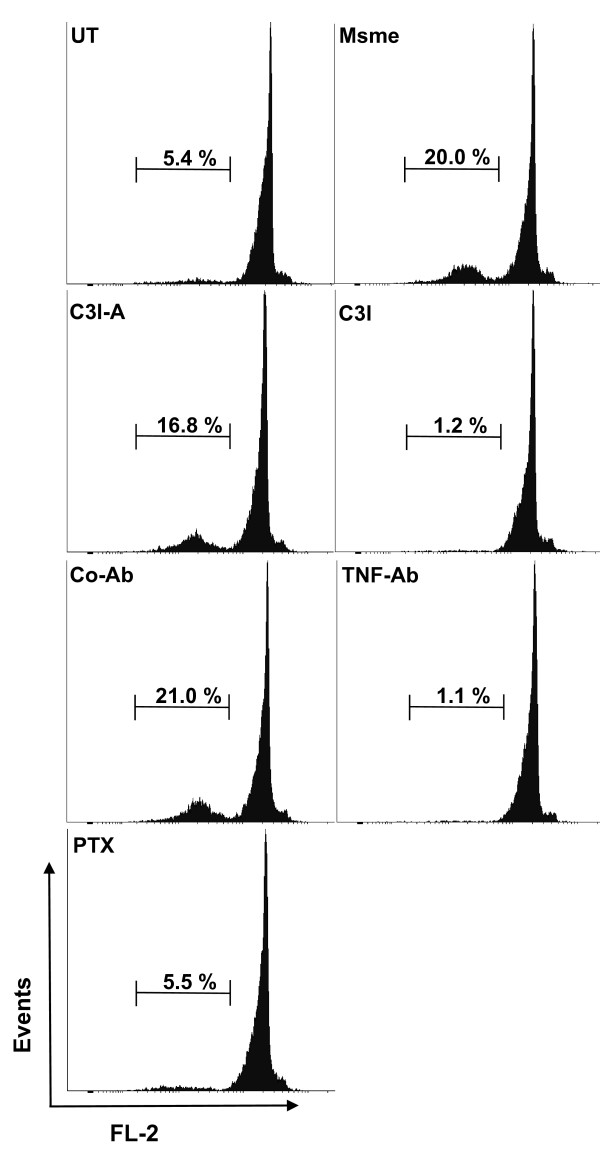
**Macrophage apoptosis induction by a non-pathogenic mycoabcterium is caspase-3 and TNF-dependent**. BMDMs from BALB/c mice were left untreated and uninfected (UT) or infected with *M. smegmatis *and then either left in medium (Msme) or treated with caspase-3 inhibitor (C3I), nonspecific chemical analog (C3I-A) neutralizing TNF antibody (TNF-Ab), nonspecific control Ab (Co-Ab) and TNF synthesis inhibitor pentoxifylline (PTX). The percentage of apoptotic cells out of 10,000 total cells was determined after 20 h using the hypodiploid PI flow cytometry assay and a representative histogram of two independent experiments performed in duplicates is shown.

We showed that non-pathogenic mycobacteria induce a strong apoptotic response and TNF secretion in BALB/c macrophages (Figures 1B and 5A) when compared to facultative-pathogenic mycobacteria. Apoptosis of eukaryotic cells can follow either a caspase-dependent or caspase-independent pathway. All caspase-dependent pathways lead to activation of effector caspase-3/6/7 [33]. In order to determine which pathway was involved in the macrophage apoptotic response to non-pathogenic mycobacterial infection, we pretreated BALB/c BMDMs with caspase-3 inhibitor, TNF neutralizing antibody, pentoxifylline (a chemical inhibitor of TNF synthesis), the appropriate controls, or left the cells untreated then infected them with *M. smegmatis *at MOI of 10:1 for 2 hours. The cells were then incubated in media with gentamycin for an additional 20 hours. Host cell apoptosis was determined on 10,000 cells using the hypodiploid flow cytometry assay. In a representative experiment, cells treated with the caspase-3 inhibitor showed a significant decrease in apoptosis (1.2%) when compared to the untreated *M. smegmatis *infected control (20.0%) and to cells treated with an inactive chemical analogue of the caspase-3 inhibitor (16.8%) (Figure 6). TNF neutralizing antibody (1.1%) or pentoxifylline treated cells (5.5%) also showed a very significant decrease in apoptosis compared to the untreated (20.0%) or nonspecific antibody treated cells (21.0%) (Figure 6). These results demonstrate that that apoptosis of BMDMs induced by nonpathogenic mycobacteria is dependent upon TNF secretion and caspase-3 activation.

The increased cytokine secretion by macrophages upon infection with non-pathogenic *M. smegmatis *versus facultative-pathogenic *M. avium *has been demonstrated in human and murine macrophages and human neutrophils [15,34,35]. Our study builds upon these previous results by extending the analysis to include several non-pathogenic versus several facultative-pathogenic mycobacteria. We underscore that the strong pro-inflammatory response elicited by macrophage might be a more general characteristic of non-pathogenic mycobacteria. The increase of TNF secretion induced by *M. smegmatis *in murine BMDM is dependent upon stimulation of the cAMP/protein kinase A pathway which results in prolonged ERK1/2 activation[15]. Furthermore, *M. smegmatis *infection leads to increase in TNF and NOS2 promoter activity but not infection with *M. avium *[15,36]. The present study also extends upon these previous findings by linking the increase in TNF secretion to pro-apoptotic capacity of the non-pathogenic mycobacteria (Figure 6) and characterizing this apoptosis pathway as being caspase-dependent (Figure 6).

### Non-pathogenic mycobacteria do not induce apoptosis in C57Bl/6 BMDM

**Figure 7:**
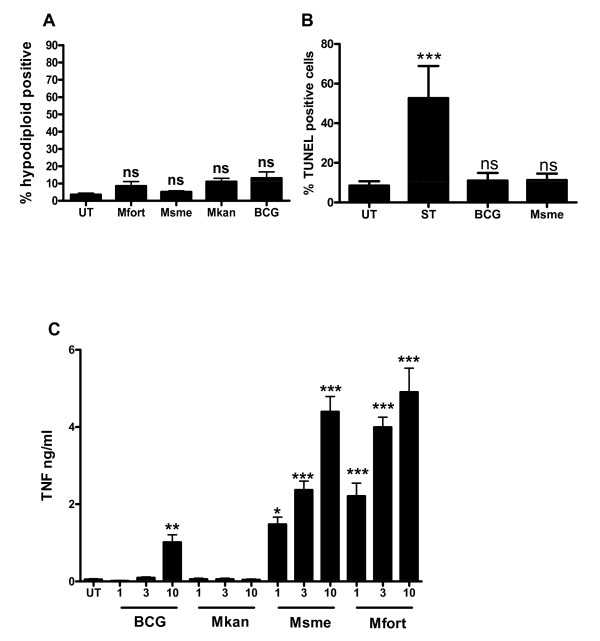
**Mycobacteria do not induce rapid apoptosis in BMDM originating from C57Bl/6 mice**. **A**. Differentiated C57Bl/6 BMDMs were infected at an MOI of 10:1 with *M. smegmatis *(Msme), *M. fortuitum *(Mfort), *M. kansasii *(Mkan), *M. bovis *BCG or left untreated (UT). The percentage of apoptotic cells was determined using a propidium iodide based staining protocol to detect the population of hypodiploid cells via flow cytometry at 20 h after infection. **B**. C57Bl/6 BMDMs were infected as in A. or incubated with staurosporine (ST) and the amount of apoptosis was detected using TUNEL staining and flow cytometry analysis. **C**. Macrophages were infected at MOIs of 1:1, 3:1, and 10:1 with *M. smegmatis *(Msme), *M. fortuitum *(Mfort), *M. kansasii *(Mkan), *M. bovis *BCG, or left untreated (UT). Culture supernatants of triplicate wells were collected after 20 h and the amounts of secreted TNF was determined using ELISA. In A. and B. the data shown is the mean and standard deviation of three independent experiments. In C. the values are the mean and standard deviation of triplicate readings of one experiment and they are representative of three independent experiments.

We demonstrated that non-pathogenic mycobacteria induce a strong apoptotic response and TNF secretion in BALB/c macrophages (Figures 1 and 5) when compared to facultative pathogenic mycobacteria. We also demonstrated that TNF plays a role in this apoptotic response (Figure 6). We therefore intended to further clarify the role of TNF by using TNF knock-out mice. Nevertheless, to our surprise we determined that BMDMs from C57Bl/6 wild-type mice, which is the genetic background of the TNF deficient mice, did not undergo apoptosis upon infection with non- and facultative-pathogenic mycobacteria using two different apoptosis detection assays (p > 0.05; Figure 7A and 7B). Interestingly, non-pathogenic mycobacteria still induced a significant increase of TNF secretion in the C57Bl/6 macrophages (Figure 7C). Indeed, they secreted around 5 fold more TNF when infected with *M. smegmatis *and *M. fortuitum *compared to infections with BCG and *M. kansasii*, the latter of which did not induce the secretion of any detectable amounts of TNF (Figure 7C).

These results demonstrate that the apoptotic response upon infection with non-pathogenic mycobacteria is dependent on the genotype of the host. The total amount of TNF secreted after *M. smegmatis *infection is reduced in C57Bl/6 versus BALB/c BMDMs (Figures 5A and 7C). For example at an MOI of 10:1 *M. smegmatis *induces 16.7 ± 2.7 ng/ml in BALB/c macrophages but only 4.4 ± 0.7 ng/ml in C57Bl/6 (p < 0.01). This could be interpreted as evidence for the role of decreased TNF secretion in the absence of *M. smegmatis *induced apoptosis of C57Bl/6 BMDMs. Nevertheless, infection of BMDMs of either mouse strain by *M. fortuitum *results in very similar induction of TNF secretion of 6.2 ± 2.0 ng/ml and 4.9 ± 1.1ng/ml in BALB/c and C57Bl/6, respectively (p > 0.05; Figures 5A and 7C) but still *M. fortuitum *just like *M. smegmatis *only induces apoptosis in BALB/c BMDMs but not C57Bl/6 cells (Figures 1B and 7A). We hypothesize thus that a certain amount of TNF secretion is necessary but not sufficient to mediate apoptosis induction of BMDMs. In a recent study we demonstrated a similar dissociation between induction of TNF secretion and host cell apoptosis[7]. A pro-apoptotic Mtb mutant still induced TNF secretion but not host cell apoptosis in BMDMs lacking functional phagocyte NADPH oxidase (NOX2). It is thus intriguing to speculate that BALB/c and C57Bl/6 NOX2 enzymes react differently upon phagocytosis with non-pathogenic mycobacteria with the former inducing a stronger, prolonged activity resulting in a greater increase in ROS. It could be that this increase in ROS would tip the balance of the autocrine TNF-signaling towards apoptosis via increased JNK activation[37].

### Differences in apoptosis induced by facultative-pathogenic and non-pathogenic mycobacteria in BALB/c and C57BL/6 dendritic cells

**Figure 8:**
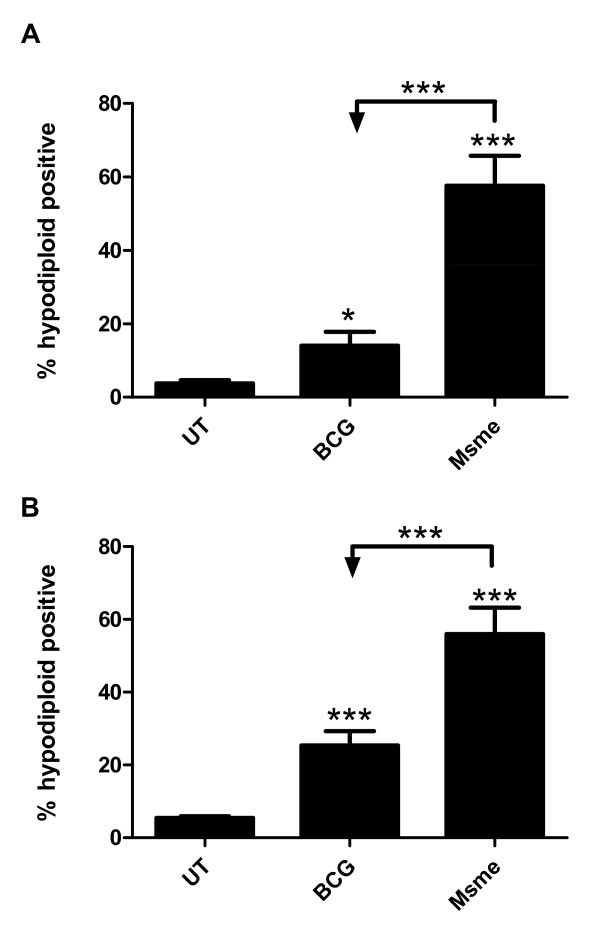
**Differences in apoptosis induced by facultative-pathogenic and non-pathogenic mycobacteria in BALB/c and C57Bl/6 dendritic cells**. C57Bl/6 (**A**) and BALB/c (**B**) bone marrowderived dendritic cells (BMDD) were infected at an MOI of 10:1 with *M. smegmatis *(Msme), *M. bovis *BCG or left untreated (UT). After 2 h cells were washed and incubated in infection media with 100 μg/ml gentamycin for an additional 20 h. The percentage of hypodiploid cells of a total of 10,000 cells was determined using the flow cytometry. The values of the mean and standard deviation of three independent experiments are shown.

**Figure 9:**
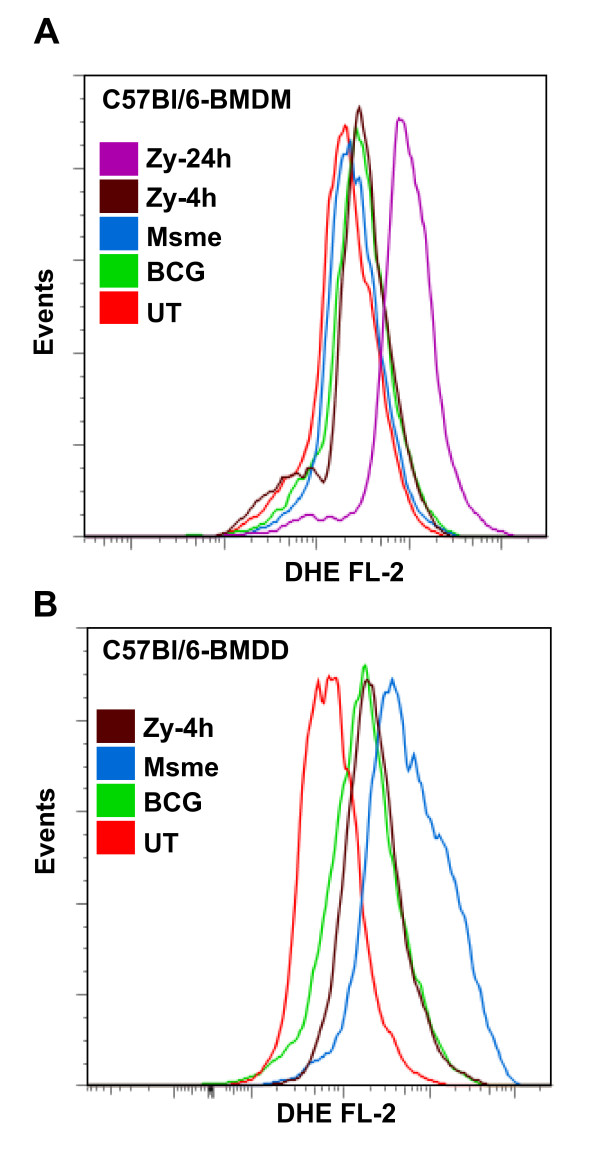
**Differences in ROS response to mycobacterial infection between C57Bl/6 macrophages and dendritic cells**. Cells were infected as described in figure 8 and ROS were detected 2 h after infection using dihydroethidium (DHE). **A**. BMDM or **B**. BMDD left untreated (UT), infected with BCG, *M. smegmatis *(Msme) or incubated with opsonized zymosan particles for 4 h or 24 h. The increase in DHE mediated fluorescence (FL-2) was analyzed by flow cytometry of 10,000 total cells. A representative of three independent experiments is shown.

*M. tuberculosis *resides primarily in alveolar macrophages of infected humans. Nevertheless, at least in the lungs of infected mice, a large percentage of *M. tuberculosis *infected cells were found to be dendritic cells [38]. Consequently, we examined whether the difference in the apoptotic response between non-pathogenic mycobacteria and facultative-pathogenic mycobacteria observed in macrophages also manifests itself in bone-marrow-derived dendritic cells (BMDD). Thus BALB/c and C57BL/6 BMDDs were infected with GFP-expressing *M. smegmatis *and BCG strains for two hours, then washed and incubated in media with gentamycin for an additional 20 hours. The rate of infection was similar across all conditions and cells as determined by flow cytometry (GFP fluorescence intensity shifts) and colony forming units on agar plates (data not shown). The number of hypodiploid positive cells was quantified using the PI-based flow cytometry assay described before. *M. smegmatis *infected C57BL/6 and BALB/c dendritic cells showed a significant increase in apoptosis (about 60% in both) when compared to BCG and uninfected cells (p < 0.0001; Figure 8A and 8B). Interestingly, in contrast to BMDMs in BMDDs the facultative-mycobacteria BCG induced a significant increase in apoptosis after one day of infection of about 15% for C57Bl/6 and 25% for BALB/c compared to about 5% in untreated cells (p < 0.0001; Figure 8A and 8B). Our results suggest that BMDDs are inherently more susceptible for undergoing apoptosis upon infection with facultative mycobacteria than macrophages in BALB/c (compare Figures 1B and 8B). They also indicate that there is a profound difference between bone marrow-derived macrophages and dendritic cells in C57Bl/6 mice in regard to apoptosis induction upon infection with non-pathogenic mycobacteria (compare Figures 7A and 8A). This difference could be due to the inherently increased activity of NOX2 enzyme complex in dendritic cells when compared to macrophages [39]. NOX2 in dendritic cells is thought to keep the phagosome at a more neutral pH in order to facilitate generation antigenic peptides for cross presentation [39]. One of the consequences of increase NOX2 activity is an accumulation of reactive oxygen species (ROS) and increases in ROS levels have been shown to shift the balance of TNF-R1 signaling in favor of JNK activation and the induction of apoptosis [32,37]. In order to address the potential role of ROS mediated apoptosis induction in C57Bl/6 derived BMDMs and BMDDs, cells were infected as described before and the amount of ROS was detected using dihydroethidium (DHE) and quantified by flow cytometry (Figure 9). Interestingly, in BMDMs only the zymosan control induced any increase in ROS levels after 24 h of incubation (Figure 9A). In contrast, in the dendritic cells *M. smegmatis *infection induced an important accumulation of ROS when compared to zymosan and BCG infected cells (Figure 9B). These results support the argument that dendritic cells are more susceptible to infection-induced apoptosis due to their capacity to generate high levels of ROS due to sustained NOX2 activity when compared to the rapid induction and inactivation of NOX2 in macrophages[39].

## Conclusions

We hypothesized that the attenuation of non-pathogenic versus facultative-pathogenic mycobacteria could be explained in part by their strong induction of an innate immune response. Indeed, here we demonstrate that two representative strains of non-pathogenic mycobacterial species induce a stronger inflammatory response as measured by the cytokines TNF and IL-12. They also induce an increased apoptotic response in BMDMs and BMDDs. The PI-LAM and Man-LAM cell wall components of non-pathogenic and facultative-pathogenic mycobacteria, respectively, were analyzed. They could be a reason for the increased innate immune response since PI-LAM induces increased cytokine secretion and apoptosis response when compared to Man-LAM. We propose that the different mycobacterial species can be characterized by the following three functional groups: 1) Nonpathogenic which have no mechanisms to inhibit immune responses and contain a lot of PAMPs to induce a response, 2) facultative-pathogenic mycobacteria have few if any mechanisms to inhibit host cell immune responses but have evolved to mask some of their PAMP so they do not induce a strong innate response and finally 3) highly adapted virulent mycobacteria mask their PAMP and have mechanisms to inhibit host immune responses.

## Methods

### Bacteria

*M. smegmatis *strain (mc^2 ^155) was obtained from Dr. William Jacobs Jr., and *M. fortuitum *strain (ATCC 6841) and *M. kansasii *strain Hauduroy (ATCC 12478) were obtained from the American Type Culture Collection http://www.atcc.org. *M. bovis *BCG Pasteur strain was obtained from the Trudeau Culture Collection (Saranac Lake, New York, United States). GFF-expressing BCG and *M. smegmatis *were generated by subcloning the enhanced GFP gene (Clonetech, http://www.clonetech.com) into the mycobacterial episomal expression vector pMV261. The resulting plasmid (pYU921) was transfected into competent cells by electroporation as previously described (Snapper et.al,). *M. smegmatis *was cultured in LB broth with 0.5% glycerol, 0.5% dextrose, and 0.05% TWEEN-80. *M. fortuitum, M. kansasii*, and *M. bovis *BCG were cultured in 7H9 broth with 0.5% glycerol, 0.5% dextrose, and 0.05% TWEEN-80, and 10% ADC enrichment. For selective media, 40 μg/ml kanamycin was added.

### Bone marrow-derived macrophages and dendritic cells

Four to six weeks old BALB/c or C57BL/6 mice were obtained from the National Cancer Institute. Mice were used before twelve weeks of age and sacrificed by CO_2 _asphyxiation followed by cervical dislocation in accordance with IACUC approved protocols. The anterior limbs were flushed with DMEM supplemented with 2% fetal calf serum. Flushed bone marrow cells were then pelleted and treated with 1× red blood cells lysis buffer (eBiosciences) for 10 minutes then washed with 1× phosphate buffered saline. For macrophage differentiation, Cells were then plated on Petri dishes in DMEM medium supplemented with 10% heat inactivated fetal calf serum, 15% L929 cell supernatant, 1% Penicillin/Streptomycin, and 2% HEPES then incubated at 37°C/5% CO_2_. Cells were supplemented with additional medium on day three. On day 7, all non-adherent cells were washed off and the remaining adherent bone marrow-derived macrophages were seeded on appropriate plates for infection.

To derive dendritic cells, cells were incubated in medium as described for macrophages but containing 20 ng/ml murine GM-CSF (Peprotech) instead of L929 supernatant. 1 × 10^6 ^cells/well were added to 6 well plates containing 2.5 ml medium and an additional 2.5 ml medium/well was added on days 3, 6, and 9. All non-adherent dendritic cells were collected and seeded on appropriate plates for infection.

### Cell cultures conditions and infection

For the apoptosis assays, 5 × 10^5 ^bone marrow-derived macrophages or dendritic cells in DMEM supplemented with 10% fetal calf serum, and 2% HEPES (infection media) were seeded on each well of a 24 well plates. Bacteria were grown to an OD_600 _ranging from 0.2 - 0.8, passed through a 26 Gauge needle 3 times and allowed to settle for 10 minutes. The infection was carried out at a multiplicity of infection (MOI) of 1:1, 3:1, and 10:1 for 2 h in duplicate wells, after which extracellular bacterial were removed by 3 washes using PBS. The cells were then incubated in DMEM infection medium supplemented with 100 μg/ml gentamycin (Invitrogen) for 20 h and the apoptosis assay was performed.

### TNF and IL-12 assays

For the TNF secretion assays, 2 × 10^5 ^bone marrow-derived macrophages in DMEM infection media were seeded onto each well of 12 well plates and infected with bacteria as indicated above. The culture supernatants were then collected 20 h after incubation in infection media supplemented with 100 μg/ml gentamycin. The amount of TNF in supernatants was then measured via ELISA (BD Bioscience). The RAW IL-12 promoter cell line was created and used to measure IL-12 p40 induction as described in great detail in our previous publication [12].

### TLR interaction assay

The Chinese hamster ovary (CHO) cells transfected with the inducible membrane protein CD25 under control of a region from the human E-selectin promoter containing nuclear fact-kB binding sites and expressing CD14 and either human Toll-like receptor 2 (TLR-2) or human TLF-4 were created as described in [28] kindly provided by Dr. D.T. Golenbock. Cells were used exactly as described previously by our group [12].

### Apoptosis assays

In most of the experiments the flow cytometry-based, hypodiploid assay was used for the detection of apoptosis after infection of bone marrow-derived macrophages and dendritic cells. Cells were collected after infection, pelleted and resuspended in propidium iodine (PI)/RNase buffer (BD Pharmingen) for 20 min and the percentage of hypodiploid positive cells was determined by flow cytometry in duplicates in the FL-2 channel at 580 nm (FACS-Calibur, BD Biosciences). The TUNEL assay was performed as suggested by the manufacturer (Roche) and described previously [8]. The apoptosis induction mediated by lipoglycanes was analyzed via AnnexinV-Alex488 (Molecular Probes) and PI double staining and flow cytometry as described previously [12].

### Caspase inhibition and TNF neutralization assays

BMDMs from BALB/c mice were treated with a pan-caspase-3/6/7 inhibitor (100 μM), caspase-3 inhibitor negative control (100 μM) (both from Calbiochem), anti murine TNF neutralizing antibody (5 μg/ml), isotype control antibody (5 μg/ml) (both from BD Bioscience), or pentoxifylline (Sigma, 100 μg/ml) for 1 h at 37°C/5% CO_2 _then infected with *M. smegmatis *at MOI 10:1 for 2 h as described above. Cells were then washed 3 times in PBS and incubated for an additional 20 h in DMEM infection media supplemented with the appropriate inhibitors and controls mentioned above and the apoptosis assay was performed.

### ROS detection assay

Reactive oxygen species in BMDM and BMDD cells were detected at 2 h post infection using the ROS sensitive dye dihydroethidium (DHE) (Invitrogen). BMDM or BMDD cells were deprived of L929 supernatant or rGM-CSF respectively 16 hrs prior to infection and maintained in cytokine free media without phenol red for the length of the experiment. Post infection, cells were washed once in HBSS and then incubated in 2 μM DHE for 15 minutes. Cells were washed 3 times with HBSS, fixed with 4% paraformaldehyde and analyzed by flow cytometry.

## Authors' contributions

AB, KV and HA designed and performed experiments and analyzed data. VB analyzed the data and wrote the manuscript. All authors approve the final manuscript.
